# Ilizarov bone transport as a treatment of congenital pseudarthrosis of the tibia: a long-term follow-up study

**DOI:** 10.1007/s11832-015-0675-7

**Published:** 2015-08-13

**Authors:** Jan Vanderstappen, Johan Lammens, Pieter Berger, Armand Laumen

**Affiliations:** Department of Orthopaedic Surgery, University of Leuven, Leuven, Belgium; Ilizarov Department, Department of Orthopaedic Surgery, University of Leuven, Leuven, Belgium; Department of Pediatric Orthopaedics, Department of Orthopaedic Surgery, University of Brussels, Brussels, Belgium

**Keywords:** Congenital pseudarthrosis of the tibia, Ilizarov bone transport, Neurofibromatosis

## Abstract

**Purpose:**

Most studies on congenital pseudarthrosis of the tibia (CPT) report on the short-term union rate and refracture rate but do not take into account the long-term outcome. This review includes patients treated with an Ilizarov bone transport, who all reached skeletal maturity. It describes long-term results and highlights any prognostic factors that could predict the final outcome.

**Methods:**

The records of patients with CPT treated with an Ilizarov bone transport in our institution were retrospectively evaluated.

**Results:**

A total of 12 consecutive patients were studied. The mean follow-up was 24.5 years (range 6–39 years). Primary consolidation was seen in ten patients (83 %). Half of these patients had a refracture. At final follow-up, eight patients experienced union and four remained un-united, of whom one had an amputation.

**Conclusions:**

The present data confirm a good primary healing rate. However, tibial union at final follow-up was only seen in 67 %, indicating that refracture is the main issue. United bone is often of inferior biological and mechanical quality, so lifetime protection with intramedullary devices, braces or a combination of both is recommended.

## Introduction

Congenital pseudarthrosis of the tibia (CPT) remains one of the most challenging problems in paediatric orthopaedic surgery due to its low incidence, unclear aetiopathogenesis and resistance to therapy. There is a definite link with neurofibromatosis type 1 (NF-1), an association already described in 1937 [1]. For non-NF patients, the aetiopathogenesis is completely unknown, but the clinical presentation is perfectly comparable and has the same reluctance to healing. Treatment often requires complete resection of the affected area and a reconstruction using intramedullary stabilisation and bone grafting, vascularised fibular transplant or Ilizarov bone transport [2]. Also, the combined use of these methods and bone morphogenetic protein 2 or 7 have been described [3–6]. Comparison of the results of different methods and even within the same technique is difficult due to variable follow-up periods, different criteria for healing and lack of long-term follow-up. This review includes a series of 12 patients treated with an Ilizarov bone transport with a long-term follow-up. It describes their final outcome and any prognostic factors contributing to the evolution and long-term result.

## Patients and methods

The records of patients with CPT treated in our institution were retrospectively evaluated. Only those patients who had been treated with an Ilizarov bone transport and had reached skeletal maturity were further studied. The treatment consisted of extensive debridement of the pseudarthrosis (a segmental resection of the pseudarthrosis site) and Ilizarov bone transport. If standard follow-up radiographs failed to demonstrate signs of bone healing after 6–10 weeks, autologous bone grafting of the docking site was performed (OP-1 in one patient). There was no intention to obtain a united fibula. The investigated items included sex, age at first visit, affected side, length of follow-up, Crawford classification, age at first surgery, outcome, number and type of surgical procedure, and age at each intervention. Also, the presence or absence of refracture was recorded, the refracture-free interval, deformities and leg length discrepancy (LLD) after the final treatment, walking aids and status of the fibula.

The outcome was classified into three groups: union, non-union or amputation. Union was radiographically defined as restoration of cortical integrity with bridging callus across minimally three visible cortices on anteroposterior and lateral views, and clinically no pain at the fracture site on palpation and full weight-bearing.

Residual deformities were evaluated on radiographs in the mediolateral and anteroposterior planes by measuring respectively the lateral distal tibia angle (LDTA) and the anterior distal tibia angle (ADTA). Fibula status was radiographically determined as union, non-union or resection.

## Results

### Patient demographics (Table 1)

A total of 12 consecutive patients (eight male, four female) with CPT treated between 1974 and 2007 at our institution were studied. The right/left ratio of the affected side was 7/5. The mean follow-up was 24.5 years (range 6–39 years). The average age at first visit was 4.7 years (range 0–16 years). According to Crawford’s classification, two tibiae showed a type 1 lesion (sclerosis). There was twice a type 3 cystic lesion and on four occasions there was a type 4 frank pseudarthrosis. From four patients, there were no original radiographs available allowing any classification. The pseudarthrosis site was in the distal third of the tibia in all patients except in one (middle third).

**Table 1:** Patient demographics. Concerning the level of the pseudarthrosis site, 2/3 indicates the middle third of the tibia and 3/3 the distal third

Patient number	Sex	Side	Age (years)	Age at first visit (years)	Length of follow-up (years)	Crawford classification	Level	NF
1	M	R	21	9	10	1	3/3	No
2	M	L	20	14	6	Unknown	3/3	Yes
3	M	L	29	16	13	4	3/3	No
4	M	L	40	1	39	Unknown	3/3	Yes
5	F	L	43	4	39	4	3/3	No
6	M	R	27	0	27	4	3/3	No
7	F	R	30	9	21	3	3/3	No
8	M	L	32	0	32	Unknown	3/3	Yes
9	M	R	28	2	26	3	3/3	Yes
10	F	R	33	1	32	Unknown	2/3	Yes
11	M	R	26	0	25	1	3/3	No
12	F	R	24	0	24	4	3/3	No

### Surgical procedures (Table 2)

The average age at the first intervention was 5.8 years (range 1–17 years). The number of procedures, including previous and additional surgeries, was a minimum of three and a maximum of 12 per patient in the total of almost 90 surgeries in 12 patients. Sixty of these surgical procedures were performed to obtain union, 17 to correct malalignment or shortening, six for relief of ankle pain (tibiotalar arthrodesis) and six for revision after complications such as infection or loosening of pins. Bone transport was performed in 20 surgeries, Ilizarov circular frame fixation and compression in 19, intramedullary pinning in five and intramedullary nailing in two surgeries. Autologous bone grafting was used 11 times, bone morphogenetic protein 7 once and segmental fibular resection twice.

**Table 2:** Age at first surgical procedure and number of surgical procedures. The numbers in parentheses are the numbers of procedures performed prior to the index operation

Patient number	Age at first surgical procedure (years)	Number of surgical procedures
1	9	4 (0)
2	17	4 (0)
3	16	10 (3)
4	1	6 (1)
5	4	>3 (>1)
6	1	12 (9)
7	9	10 (1)
8	2	11 (5)
9	4	12 (4)
10	2	3 (2)
11	1	7 (2)
12	4	5 (0)

Twenty-eight interventions were performed prior to the index operation. Ilizarov circular frame fixation and compression was done 11 times, intramedullary nailing twice, intramedullary pinning five times, autologous bone grafting four times, corrective osteotomy twice, Ilizarov lengthening three times and a pantalar arthrodesis once.

### Outcome (Table 3)

At final follow-up, in eight patients (67 %), the tibia healed and four were left un-united. The average age at the first surgical procedure was 4.3 years (range 1–9 years) and 9 years (range 1–17 years) respectively. The age at final union was, on average, 16.5 years (range 9–36 years).

**Table 3:** Outcome

Patient number	Outcome	Refracture	Refracture-free interval (years)	Status tibiotalar (TT) and subtalar (ST) joint	Status fibula
1	Union	Yes	4	TT fusion	Non-union
2	Non-union	No			Non-union
3	Non-union	Yes	0.25	TT + ST fusion	Non-union
4	Union	No		TT fusion	Non-union
5	Union	Yes	Unknown		Non-union
6	Amputation	Yes	18	Prior TT + ST fusion	Amputation
7	Union	No		TT fusion	Non-union
8	Union	No		TT fusion	Non-union
9	Union	Yes	2		Non-union
10	Non-union	No			Non-union
11	Union	No		TT fusion	Resected
12	Union	No			Resected

Patient number	LDTA (°)	ADTA (°)	LLD (mm)	Walking aids
1	–	–	15	Brace
2	–	–	0	AFO
3	–	–	−40	Orthopaedic shoes
4	–	–	−5	Orthopaedic shoes
5	77	96	0	Insoles
6	–	–	Amputation	Prosthesis
7	–	–	0	Insoles
8	–	–	64	None
9	79	81	−30	Orthopaedic shoes
10	–	–	−265	Prosthesis
11	–	–	−30	None
12	107	93	10	Orthopaedic shoes

**Fig. 1:**
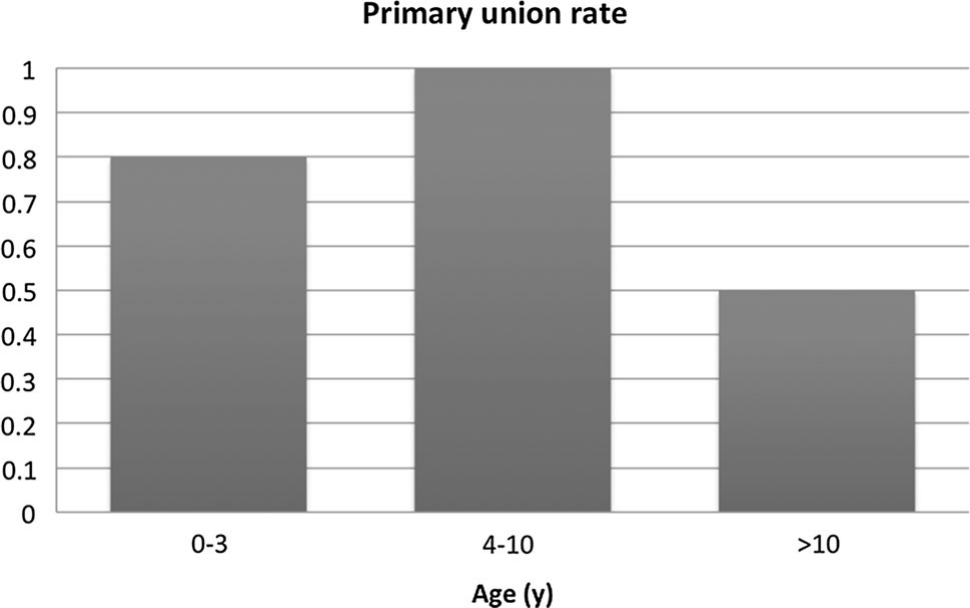
Primary union rate for the three age groups (age at first surgical procedure)

**Fig. 2:**
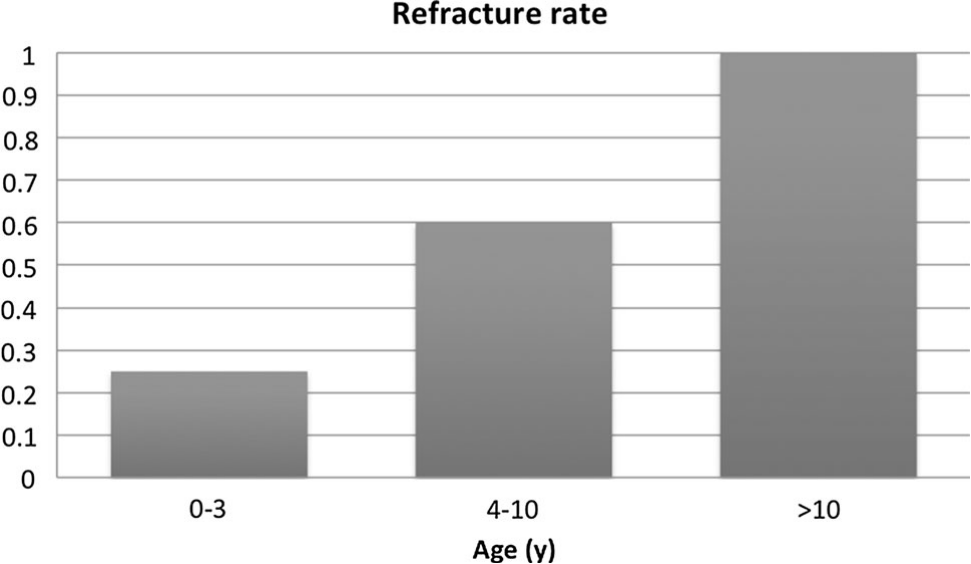
Refracture rate for the three age groups (age at first surgical procedure)

Primary consolidation, described as union after the initial treatment, was seen in ten patients (83 %). However, half of these had a refracture during follow-up. A second Ilizarov treatment again achieved successful union in three cases. In one patient, tibial union could not be achieved and in another patient, a below-the-knee amputation had to be done. The refracture-free interval was three months, two years, four years and 18 years and unknown in one patient, respectively. Figures 1 and 2 describe the primary union rate and refracture rate for the three age groups (age at first surgical procedure).

Associated fibular non-union was seen in nine cases and segmental fibular resection was performed in two patients. Five patients had a tibiotalar arthrodesis and two patients had a pantalar arthrodesis at final follow-up.

Two out of three patients with tibial union and a native ankle joint had a residual ankle valgus deformity (LDTA <80°). Both had a concomitant fibular pseudarthrosis. One patient had a residual ankle varus deformity (LDTA 107°). The mean ADTA in these patients was 90°, indicating a slight residual antecurvatum deformity.

The median residual LLD was 0 mm. One patient with a persistent non-union sustained a residual shortening of 265 mm at the end of growth.

At final follow-up, four out of eight patients with united tibiae had walking aids (one with a brace, three patients with orthopaedic shoes). Amongst the non-union patients, two had a prosthesis (one amputation, one severe shortening), one an ankle foot orthosis and one orthopaedic shoes.

## Discussion

Historically, the treatment of congenital pseudarthrosis is extremely challenging, as it is difficult to obtain and maintain a solid union. The original described techniques, such as Boyd’s onlay grafting [7], McFarland’s support graft [8] or Sofield’s rodding technique [9], were faced with multiple problems. They were abandoned in favour of more rigid intramedullary rodding procedures or Ilizarov circular frame fixation [10], often combined with extensive autologous grafting. However, due to the extent of the affected bone, large resections often create large defects to be reconstructed. Two methods can adequately deal with that problem, i.e. the vascularised fibular graft and Ilizarov’s bone transport method, but they do not always guarantee a solid union.

In the current study, the final long-term healing rate with follow-up till many years after reaching skeletal maturity was 67 %. At first sight, this is perfectly comparable to the results of the Norwegian group of Horn et al., who reported 66 % of healing with the Ilizarov technique in a group of 22 patients. However, this was primary healing in a heterogeneous group (15 Ilizarov treatments and six bone transports) and many patients refractured afterwards, making the ultimate outcome less clear [11]. In their large multicenter survey made by the European Paediatric Orthopaedic Society (EPOS)—in which the results of some of these patients are also included—Grill et al. reported a healing rate of 59.20 % in 20 segmental bone transports [12]. Guidera et al. described a union rate of 82 % in 11 patients (nine bone transports) but had a short follow-up (three years) and no union criteria [13]. A more recent study of 12 patients treated with a bone transport reported excellent results in eight patients, good in three and poor in one. However, no further details were given and the mean follow-up was five years [14]. Boero et al. had a healing rate of 65 % in 21 patients with a mean follow-up period of two years (three bone transports, none healed) and Cho et al. achieved union in 22 out of 23 patients, with a mean follow-up of 9.2 years (four bone transports) [15, 16].

However, in our study, four out of eight patients in the union group had walking aids (three orthopaedic shoes and one a protective brace). On the other hand, these patients were functionally well and ambulated pain-free without crutches or a walking stick. The outcome is considered good in one-third, acceptable in one-third and bad in one-third.

It is clear that the key strength of this study is the long-term follow-up (even up to 40 years!) in a homogeneous group of patients treated with a bone transport.

Even if a union seems to be obtained, a refracture within the first couple of years is not uncommon, as was observed in five out of ten patients (50 %) who had a healed tibia after the initial treatment. The refracture-free interval was, on average, 6.1 years. One adult patient even sustained a refracture after 18 years, suggesting that, even after remodelling and reaching skeletal maturity, bone of normal quality cannot be guaranteed. This might be an argument for protective bracing or prophylactic rodding of the tibia. Novel insights in cellular pathways showed that a mutation in the NF-1 gene results in a loss of neurofibromin activity, which disturbs the osteoblast activity [17]. This may suggest that NF patients are more prone to late refracture. This is consistent with some reports in the literature [18–20], although some studies suggest that NF has no influence on the final outcome [21–23]. In the present study, four out of five patients who sustained a refracture were NF negative.

Many studies have drawn attention to the age at which a first surgical procedure has to be initiated. Almost half a century ago, Boyd and Sage advocated surgery as early as possible, and this point of view is still defended by some authors [22, 24, 25]. However, these authors found that intramedullary nailing with bone grafts obtains better fusion rates and has less influence on the growth in children under the age of 3 years. On the other hand, Grill et al. reported an overall healing rate of only 64 % in children under 3 years old, whereas the age category between 6 and 9 years obtained a union in 92 % of cases [12]. In our study, the primary healing rate was 80 % (4/5) when the first surgery was done between 0 and 3 years old, 100 % (5/5) between 4 and 10 years old, and 50 % (1/2) after the age of 10 years. The refracture rates in these age groups were 25 % (1/4), 60 % (3/5) and 100 % (1/1), respectively. However, these groups are small and more studies with larger cohorts would be useful. Nevertheless, based on Grill et al.’s results and also supported by the studies of Ghanem et al. and Boero et al. [12, 15, 26], who demonstrated that the Ilizarov method in very young children was associated with low success rates, we prefer nowadays to postpone surgery till after the age of 4 years. On the contrary, it seems that beginning surgical treatment at an older age, such as around 10 years old, should also be avoided. Moreover, disuse atrophy of the bone and deformity of the ankle joint will be aggravated. In this study, both patients for whom the surgical treatment was started after the age of 10 years old did not obtain union at final follow-up. Another interesting finding is that the number of surgical procedures does not seem to influence the final outcome, as union was even obtained in patients after multiple surgeries.

The nature of the pathology and its complex treatment often lead to the development of deformities and leg length discrepancies. The most frequent deformity is a valgus ankle [12]. Valgus problems are thought to be associated with insufficient lateral buttress provided by the distal fibula. This is in accordance with our observations, which showed that two out of three patients had an LDTA of less than 80° and that both had a non-united fibula.

Severe deformities also often necessitate tibiotalar and/or subtalar arthrodesis. Theoretically, this could lead to increased stress on the pseudarthrosis site and subsequent refracture, but this could not be shown in our series, as four out of seven patients with an arthrodesis united without a previous refracture.

Residual LLD following successful union was not a major problem in our study. We observed a median LLD of 0 mm, indicating that the Ilizarov method is a very reliable method regarding the treatment of residual limb shortening after multiple procedures.

The importance of fibular stabilisation in the treatment of CPT has been emphasised by several authors. As previously mentioned, persistent fibular non-union favours valgus deformities. Moreover, fibular pseudarthrosis was also found to be related to failure of tibial union [27]. The findings of the current study could not support this statement, as all patients had a resected or non-united fibula. As previously mentioned, there was no intention to obtain fibular union.

Many patients require multiple revision surgeries and, even after frequent interventions, limb function can be severely compromised. Amputation for intractable CPT is not uncommon and should be considered for patients with a poor functional result after previous treatments, with very restricted walking ability, who have a good chance for improvement in case of correct prosthetic fitting and absence of stump problems. In a comparative study of gait and function between healed patients after multiple surgeries and those with an amputation, Karol et al. did not find a difference in the efficiency of gait in one of the two groups [28].

At final evaluation of our patient group, amputation was only performed once, as it was estimated throughout the treatment that this would have no benefit regarding their function. In general, we would only recommend amputation if the expected result would surpass the functional outcome after multiple surgeries.

The present data confirm a primary healing rate of 83 %. However, tibial union at final follow-up was only seen in 67 %, indicating that refracture is the main issue. United bone is often of inferior biological and mechanical quality (small docking site, sclerotic bone with no medullary cavity, non-united fibula), so lifetime protection with intramedullary devices, braces or a combination of both is recommended.
